# *Strongyloides*—An uncommon cause of eosinophilia whilst on durvalumab

**DOI:** 10.1002/cnr2.1682

**Published:** 2022-07-18

**Authors:** Gaik Tin Quah, Gillian Blanchard, Neil Miller, Paul Wilson, Ina Nordman

**Affiliations:** ^1^ Department of Medical Oncology, Calvary Mater Newcastle Waratah Australia; ^2^ Department of Infectious Diseases Calvary Mater Newcastle Waratah Australia

**Keywords:** durvalumab, eosinophilia, immune checkpoint inhibitors, immunotherapy, *Strongyloides*

## Abstract

**Background:**

In malignancy, eosinophils have been shown to play an important role in the tumour micro‐environment. Increasingly, development of eosinophilia with immune checkpoint inhibitor (ICI) use is thought to be predictive of prognosis and development of immune‐related adverse events. However, there are many other causes for developing eosinophilia which can contribute to the difficulties in diagnosis and management.

**Case:**

Here, we present a case of *Strongyloides* parasitic infection as an uncommon differential for eosinophilia in a patient with lung cancer receiving a PDL‐1 ICI, durvalumab, in Australia.

**Conclusion:**

This case highlights the complexities exploring the multiple potential causes of eosinophilia and the subsequent management, to allow safe continuation of ICI.

## 1 CASE REPORT

We present a case of a 64‐year‐old male, diagnosed in August 2020 with stage 3A left non‐small cell lung cancer (NSCLC). A positron emission tomography (PET) scan at diagnosis showed a large left hilar mass, with small mediastinal nodes. Endobronchial ultrasound (EBUS) guided biopsy confirmed carcinoma for which immunohistochemistry (IHC) was positive for P40 and negative for thyroid transcription factor 1 (TTF‐1), consistent with squamous cell carcinoma. Programmed death‐ligand 1 (PDL1) was low at 20%. He received 6 weeks of definitive concurrent chemo‐radiotherapy with weekly carboplatin and paclitaxel. He developed a mild infusion reaction to the last two doses of paclitaxel which required steroid pre‐medications. Pathology workup including eosinophil count was normal at this point. A PET scan and brain magnetic resonance imaging scan at completion of chemo‐radiotherapy showed good response to treatment. He then commenced two weekly maintenance therapy with durvalumab, an ICI, in October 2020, as per the PACIFIC trial.^1^

After the first dose of durvalumab, he was noted to have eosinophilia of 5.4 × 10^9^/L (<0.5) on routine blood work‐up. He was well, and asymptomatic. Specifically there were no symptoms to suggest toxicities from durvalumab. Therefore, he proceeded to cycles 2 and 3 of durvalumab. The eosinophil count continued to rise, and 4 weeks later, post cycle 3 of durvalumab, peaked at 12.0 × 10^9^/L. He remained clinically well. Further investigations were undertaken to determine the cause for eosinophilia. Immunoglobulin E (IgE) levels were markedly elevated at 4573 IU/ml (<100) and tryptase levels were also elevated at 24.3 ug/L (<11). An important differential of systemic mastocytosis was thought to be less likely as the associated proto‐oncogene c‐KIT mutation was negative. Fluorescent in situ hybridization (FISH) testing to look for mutations associated with hypereosinophilic syndromes^2^ were negative. Haematology opinion was sought and the eosinophilia was initially thought to be related to durvalumab.

On closer review, the patient disclosed that following the third cycle of durvalumab he had developed diarrhoea associated with crampy abdominal pains. However, his diarrhoea settled rapidly with loperamide (standard therapy in grade 1 durvalumab‐induced diarrhoea); in view of this, ICI enterocolitis was felt to be unlikely.

*Strongyloides* serology was done as part of the workup for eosinophilia. Surprisingly, the results were strongly positive at 1.74 (<0.80). The patient lived on a farm and worked as a painter in a rural town in New South Wales, Australia. He had no previous history of a known *Strongyloides* infection and had no known risk factors. He was not of Indigenous background and had not lived in or travelled to an endemic area. Stool microscopy and culture (MCS) as well as polymerase reaction testing was negative for parasites, including *Strongyloides*. An opinion from infectious diseases team was sought, and he was commenced on the antiparasitic agent ivermectin 200 mcg/kg on days 1, 2, 15 and 16. After two doses of ivermectin, the eosinophil count dropped rapidly to 1.2 × 10^9^/L (<0.5) and to 0.9 × 10^9^ (<0.5) after completion of the ivermectin course. IgE levels remained elevated at 3991 kU/L (<214).

Discussions were held with the patient regarding continuing durvalumab checkpoint inhibitor treatment, as the diagnosis of strongyloidiasis did not completely exclude the co‐existence of eosinophilia secondary to ICI use. There was also uncertainty regarding the potential increased risk of hypersensitivity reactions to durvalumab given the eosinophilia and elevated IgE. After much consideration, he consented to receive the fourth dose of durvalumab whilst an inpatient to allow for close observation in the intensive care unit afterwards. The treatment was completed without adverse events. He has since received durvalumab safely in the outpatient setting without any issues. Recent progress CT scans show stability of the lung malignancy.

## 2 DISCUSSION

In malignancy, eosinophils have been shown to infiltrate multiple tumours, either as part of the tumour microenvironment or in response to various treatment strategies.^3^ They have been shown to have direct and indirect anti‐tumour activity in vitro.^4^ The development of peripheral eosinophilia in the context of immune checkpoint inhibitors have been examined in several retrospective studies. The absolute eosinophil count (AEC) has been shown in several studies of ICIs in metastatic melanoma to correlate with improved response rates and overall survival.^5^ Several studies have also shown a correlation between elevated AEC and development of immune‐related adverse events (irAEs).^6^ Whilst the prevalence of ICI‐eosinophilia has been reported to be as high as 22%,^6^ moderate to severe eosinophilia (>1.5 × 10^9^/L) is very uncommon at 2%.^7^ Moderate to severe eosinophilia is associated with the development of hypereosinophilic syndrome where there is end‐organ damage due to infiltration of eosinophils. This was a major concern for the patient in our case, given the significantly elevated AEC levels, which peaked at 24 times the upper limit of normal.

However, it is important to note that there are many other causes of peripheral eosinophilia, including: inflammatory, allergic and infectious conditions. These are important to exclude in the diagnostic workup before attributing eosinophilia to ICI to avoid unnecessary cessation of ICI. In the present case, the clinical history of diarrhoea and abdominal pain led to further investigations to look for infectious causes. Differential of ICI enterocolitis was considered as a potential cause for the persistent eosinophilia^8^; however, in view of a rapid resolution of diarrhoea with loperamide only, this was felt to be unlikely. Because the patient did not have a convincing travel history nor had he lived in an endemic area for *Strongyloides*, other investigations were also organised to exclude other causes of the significant eosinophilia in this patient.

Strongyloidiasis infection in humans is caused by a roundworm *Strongyloides stercoralis*. It is endemic in tropical and subtropical regions of the world where warmth, moisture and poor sanitization help its spread. Some remote indigenous communities in Australia have had prevalence of up to 60%^9^; however, it is generally uncommon outside of this setting.

The initial infection occurs when *S. stercoralis* larvae penetrate skin, and travel via the bloodstream to the heart, to enter the lungs. The larvae ascend the bronchial tree, and are swallowed by the host, where they mature into adult worms in the small intestine and then lay up to 40 eggs per day. These eggs then hatch in the lumen of the intestine, to release larvae which can penetrate the colonic wall or perianal skin and enter the body, therefore, repeating the migration cycle that establishes ongoing auto‐infection. This persistent auto‐infective cycle allows *Strongyloides* to persist for decades after the initial infection.^10^ Therefore, the host could harbour a chronic infection years after a visit to an endemic area.

The auto‐infective larvae migration can enter any organ in the body, making the signs and symptoms of this condition non‐specific and elusive. Skin, gastrointestinal and respiratory symptoms are common, but can be mild. Eosinophilia is present in the acute and chronic phases. In our patient, the faecal MCS was negative, but serology was positive. Strongyloides serology using the *Strongyloides* immunoglobulin G enzyme‐linked immunosorbent assay has a high sensitivity and specificity for chronic strongyloidiasis.^11^ Thus, in this case, it was more likely to be chronic strongyloidiasis than acute. Ivermectin is very effective in eradicating the *S. stercoralis*. Eradication can be confirmed with repeat serology at 6 months.

Patients who are immunocompromised are at risk of disseminated/hyperinfective infections. Although the use of steroids is common in the management of irAEs, it has been associated with significant mortality (>60%) in immunosuppressed patients with strongyloidiasis.^10^, ^12^

The patient required increased doses of steroids in the last 2 weeks of chemo‐radiotherapy due to infusion reactions, developing eosinophilia 6 weeks after the last dose of steroids. Exposure to steroids with hyperinfection could have contributed to the marked eosinophilia.

## 3 CONCLUSION

To the best of our knowledge, this is the first reported case of *Strongyloides* as a likely cause of significant eosinophilia whilst on durvalumab ICI therapy. We highlight the importance of a thorough workup to exclude other causes of eosinophilia whilst on ICI, to avoid premature cessation of ICI, particularly in situations where ICIs have been accepted as standard of care and have been shown to confer survival benefit, such as in this case.

Given the potentially detrimental effect of steroids in precipitating *Strongyloides* hyperinfection, and that steroids are often used in the management of irAEs including ICI‐associated eosinophilia, this remains a very important differential to exclude prior to commencing corticosteroids for eosinophilia.

In this case, we were able to identify a separate potential underlying cause for eosinophilia whilst on ICI, and commence appropriate treatment, thus allowing safe continuation of ICI therapy. However, an underlying aetiology may not always be identified, and thus the development of moderate to severe eosinophilia whilst on ICI poses challenges in management to clinicians. There is currently a paucity of literature regarding this situation, and ongoing research in this area is warranted.

## AUTHOR CONTRIBUTIONS

**Gaik Tin Quah:** Data curation (lead); writing – original draft (equal); writing – review and editing (equal). **Ina Nordman:** Supervision (lead); writing – original draft (equal); writing – review and editing (equal). **Gillian Blanchard:** Writing – review and editing (equal). **Neil Miller:** Writing – review and editing (equal). **Paul Wilson:** Writing – review and editing (equal).

## CONFLICT OF INTEREST

The authors have stated explicitly that there are no conflicts of interest in connection with this article.

## ETHICS STATEMENT

The patient provided written informed consent to be included in this study.

## Data Availability

The data that support the findings of this report are available from the corresponding author upon reasonable request.
